# Dietary Restriction Induced Longevity Is Mediated by Nuclear Receptor NHR-62 in *Caenorhabditis elegans*

**DOI:** 10.1371/journal.pgen.1003651

**Published:** 2013-07-25

**Authors:** Bree N. Heestand, Yidong Shen, Wei Liu, Daniel B. Magner, Nadia Storm, Caroline Meharg, Bianca Habermann, Adam Antebi

**Affiliations:** 1 Interdepartmental Program in Cell and Molecular Biology, Baylor College of Medicine, Houston, Texas, United States of America; 2 Max Planck Institute for Biology of Ageing, Cologne, Germany; 3 Department of Molecular and Cellular Biology, Huffington Center on Aging, Baylor College of Medicine, Houston, Texas, United States of America; 4 Institute for Global Food Security, Queen's University Belfast, Belfast, Northern Ireland, United Kingdom; 5 Cologne Excellence Cluster on Cellular Stress Responses in Aging Associated Diseases (CECAD), University of Cologne, Cologne, Germany; Stanford University Medical Center, United States of America

## Abstract

Dietary restriction (DR) extends lifespan in a wide variety of species, yet the underlying mechanisms are not well understood. Here we show that the *Caenorhabditis elegans* HNF4α-related nuclear hormone receptor NHR-62 is required for metabolic and physiologic responses associated with DR-induced longevity. *nhr-62* mediates the longevity of *eat-2* mutants, a genetic mimetic of dietary restriction, and blunts the longevity response of DR induced by bacterial food dilution at low nutrient levels. Metabolic changes associated with DR, including decreased Oil Red O staining, decreased triglyceride levels, and increased autophagy are partly reversed by mutation of *nhr-62*. Additionally, the DR fatty acid profile is altered in *nhr-62* mutants. Expression profiles reveal that several hundred genes induced by DR depend on the activity of NHR-62, including a putative lipase required for the DR response. This study provides critical evidence of nuclear hormone receptor regulation of the DR longevity response, suggesting hormonal and metabolic control of life span.

## Author Summary

Dietary restriction extends the life span of diverse species across taxa, yet the underlying mechanisms are poorly understood. In humans there are clear health benefits associated with DR such as improved serum cholesterol and lipid levels. In *Caenorhabditis elegans*, genes implicated in the TOR pathway, autophagy, protein synthesis and energy homeostasis have been shown to modulate the dietary restriction response; however their mechanism of action is still unclear. In this work, we find that the *C. elegans* nuclear hormone receptor, *nhr-62*, is required for longevity in multiple DR regimens, providing the first evidence of a nuclear receptor required for DR-induced longevity. Additionally, *nhr-62* is required for physiologic changes associated with DR, including increased autophagy and decreased levels of triglycerides, possibly through lipolysis. Moreover, *nhr-62* is responsible for regulating hundreds of genes under DR, as measured by qPCR and RNA-seq. Importantly, this work is the first to report transcriptome analysis of DR in *C. elegans* and the first to provide functional evidence that nuclear receptors are key regulators of the DR longevity response, which imply hormonal and metabolic control of longevity, possibly through alterations in fat metabolism, lipolysis, and autophagy.

## Introduction

Genetic and environmental factors can cause profound changes in organism lifespan. Genetic alterations that stimulate robust longevity across taxa include reduced insulin/IGF and TOR signaling, reduced mitochondrial function, and reduced signaling from germline stem cells [1]. One of the most pervasive environmental alterations that impacts longevity is dietary restriction (DR), a reduction in caloric uptake without malnutrition, which can increase health and life span in different organisms, including yeast, worms, flies, and rodents [2]. Whether DR induces longevity in non-human primates is still under debate, however there are clear health benefits observed [3], [4]. In humans, evidence indicates that DR lowers body temperature, insulin levels, and body fat [5], [6]. Moreover, improved serum cholesterol and lipid levels suggest a decreased risk for cardiovascular disease [7]. Conversely, overnutrition may be a risk factor for age-related disease including obesity, diabetes, heart disease, neurodegeneration, and cancer [8].

In *Caenorhabditis elegans* several different DR regimens can induce longevity. The two most widely used are dilution of bacterial food and the genetic DR mimetic *eat-2*. Dilution of bacterial food in liquid culture (BDR) was first demonstrated by Klass in 1977 to extend *C. elegans* life span, and variations of this method have been shown to enhance longevity by 20–100% [9], [10]. By this regimen, animals develop on bacterial plates *ad libitum* until adulthood, and then are shifted to liquid culture containing a dilution of bacterial food. The *eat-2* mutation affects the function of a pharyngeal acetylcholine receptor subunit, which reduces pharyngeal pumping rate and subsequent food intake throughout the life of the animal, and extends life span by 15–40% [11]. Other ways of inducing DR in *C. elegans* adults include intermittent feeding (IF), in which worms are fed every two days, dietary deprivation (DD), in which adult worms are completely removed from food, and solid DR (sDR) where bacteria is diluted on solid agar plates [12]–[14]. Curiously, life extension by these regimens requires different sets of genes, indicating that DR is not a uniform process and could result from multiple responses [11]–[16].

From genetic studies in *C. elegans*, a few key transcriptional regulators of the DR response have started to emerge. PHA-4 is a FOXA homolog required for *eat-2* and BDR induced longevity [10]. SKN-1 is an NF-E2 transcription factor required in a BDR model of DR longevity [17]. Additionally, heat shock factor and hypoxia inducible factor have been implicated in regimens resembling DD or IF [18], [19]. Reduced signaling through the nutrient sensor TOR kinase, and processes downstream of TOR that increase autophagy, reduce protein synthesis, and alter energy homeostasis may contribute to the DR response [12], [20], [21]. Nevertheless, the regulatory networks and the underlying mechanisms promoting longevity still remain unclear.

In an effort to identify regulators of DR-induced longevity, we specifically focused on nuclear hormone receptors (NHRs). Nuclear hormone receptors are transcription factors that respond to fat-soluble hormones, such as steroids and fatty acids, to directly regulate gene transcription. They are broadly implicated in the regulation of development, metabolism and homeostasis, and are well poised to coordinate events throughout the body in response to hormonal or nutritional signals [22], [23]. We hypothesized that NHRs may mediate metabolic states associated with DR, and thus could be important for DR-induced longevity. In this work we identify the HNF4α-like nuclear hormone receptor *nhr-62* as required for DR-induced metabolic and longevity responses.

## Results

### NHR-62 is Necessary for *eat-2* DR-induced Longevity

**Figure 1:**
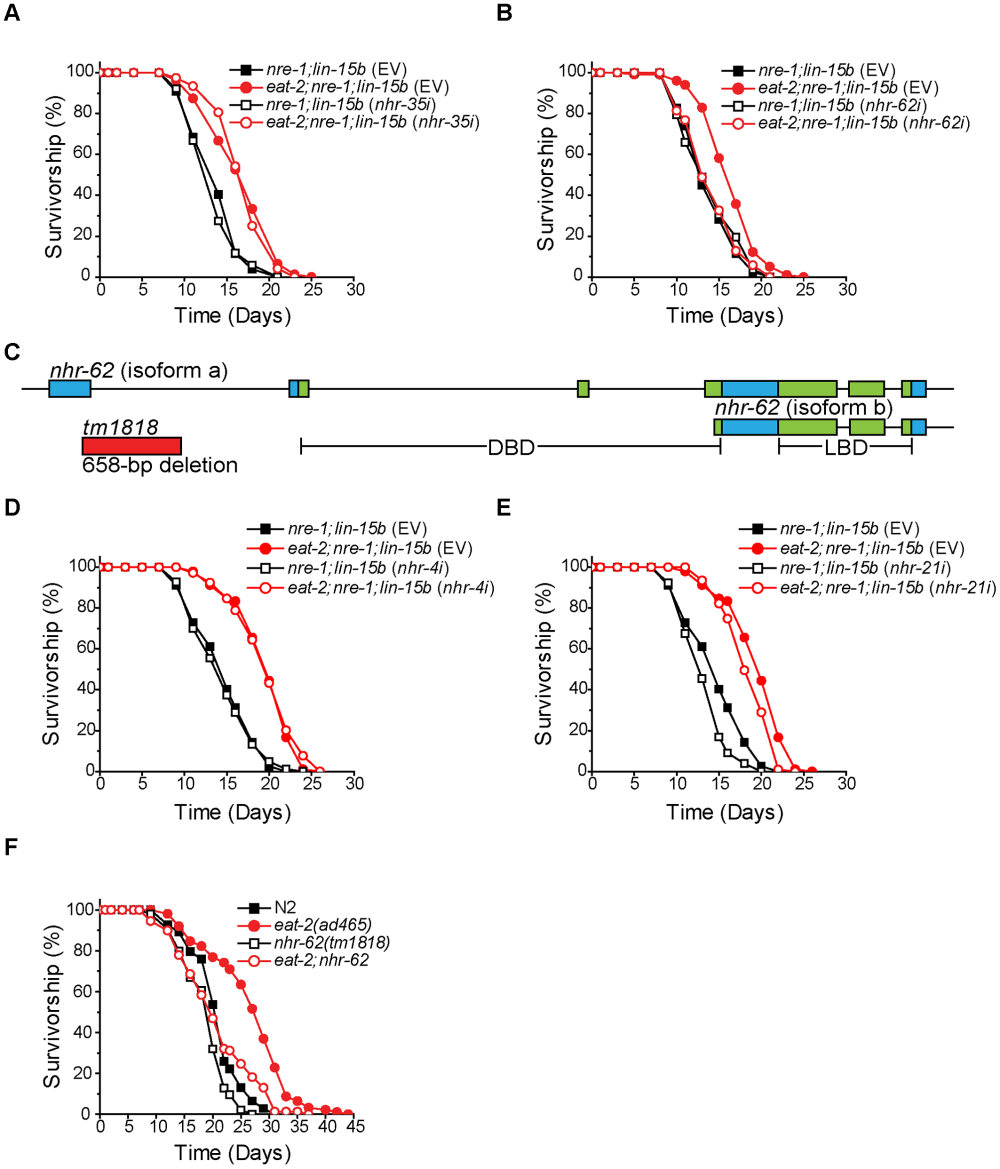
*nhr-62* mediates the longevity response to dietary restriction. (A) *eat-2;nre-1;lin-15b* worms fed either empty vector or *nhr-35* RNAi had a significant increase in lifespan compared to *ad libitum* controls (p<0.001). See Figure S1 for other NHRs. (B) *eat-2;nre-1;lin-15b* fed *nhr-62* RNAi do not have a DR-induced lifespan extension (p = 0.907). (C) Gene model for *nhr-62*. Boxes indicate exons and lines indicate introns. The DNA binding domain (DBD) and the ligand binding domain (LBD) are indicated in green. (D,E) *eat-2;nre-1;lin-15b* worms fed either empty vector, *nhr-4* or *nhr-21* RNAi had a significant increase in lifespan compared to *ad libitum* controls (p<0.001). (F) *eat-2(ad465)* had a significant increase in lifespan compared to wild-type (N2) (p<0.001). Mutation of *nhr-62* suppressed the lifespan extension of *eat-2(ad465)* worms (p<0.001). p-values calculated by the log-rank test.

To test if NHRs mediate DR-induced longevity, we performed RNAi knockdown of NHRs in animals carrying an *eat-2* mutation (a genetic DR mimetic), and screened for a loss of *eat-2*-induced longevity. We screened 246 of the 284 *C. elegans* NHRs in a genetic background more sensitive to RNAi (*eat-2;nre-1;lin-15b*). Knockdown of most NHR genes had no effect on *eat-2* longevity (*e.g.*, *nhr-35* Figure 1A). As expected, knockdown of a few NHRs substantially shortened *eat-2* and wild-type life span (*e.g.*, *nhr-49*) yet largely maintained DR-induced life span extension (Figure S1). Interestingly, we found that only knockdown of *nhr-62*, an HNF4α related NHR, suppressed the longevity of *eat-2;nre-1;lin-15b* while having little effect on the longevity of *nre-1;lin-15b* control animals (Figure 1B).

The *nhr-62* locus encodes a predicted long isoform A (515 AA) consisting of DNA- and ligand-binding domains (DBD; LBD), and a short isoform B (353 AA) consisting of only the LBD (Figure 1C). *nhr-62* shares 26–28% overall identity with vertebrate and insect HNF4α nuclear receptors across both domains. Mammalian HNF4α proteins function in many processes including lipid and glucose metabolism, and HNF4α mutations in humans are associated with maturity onset diabetes of the young type 1 [24], [25]. Similarly, the *Drosophila melanogaster* HNF4α homolog is important for lipid mobilization, fatty acid β-oxidation, and the starvation response [26]. However, 269 of the 284 NHRs in *C. elegans* represent a major expansion of the HNF4α family and are interrelated [27]. To determine specificity of the suppression of DR-induced longevity by *nhr-62*, we first compared the fraction of animals alive at day 15 between *nre-1;lin-15b* and *eat-2;nre-1;lin-15b* fed *nhr-62*'s closest homologs, *nhr-21*, *nhr-4*, *nhr-34*, and *nhr-100*, and found that these genes were not required for longevity in *eat-2;nre-1;lin-15b* mutant animals (Figure S1). Secondly, we measured the lifespan of dietarily restricted worms subjected to either empty vector RNAi or RNAi specific to the NHRs with closest homology, *nhr-21* and *nhr-4*, and again found that these genes were not required for DR-induced longevity (Figure 1D,E and Figure S1). These data suggest that *nhr-62* uniquely regulates *eat-2* DR-induced life span extension in *C. elegans*.

To validate our RNAi results, we obtained the deletion allele, *nhr-62(tm1818)*, which removes a 658 bp region, including part of exon one, and results in an immediate stop codon, thus removing the DBD of the receptor. Although *nhr-62(tm1818)* is likely a strong loss-of-function allele, it may not be null because the predicted B isoform containing the LBD only is intact. We introduced this mutation into *eat-2(ad465)* animals and measured the lifespan of the double mutant. Similar to RNAi, *nhr-62(tm1818)* significantly suppressed the lifespan of *eat-2(ad465)* animals. Importantly, the *nhr-62(tm1818)* mutant had little or no effect on wild-type (N2), suggesting that *nhr-62* does not suppress *eat-2* longevity through general sickness (Figure 1F). Though *nhr-62(tm1818)* consistently suppressed *eat-2(ad465)* lifespan, suppression was not always complete, suggesting other activities still contribute to the response.

**Figure 2:**
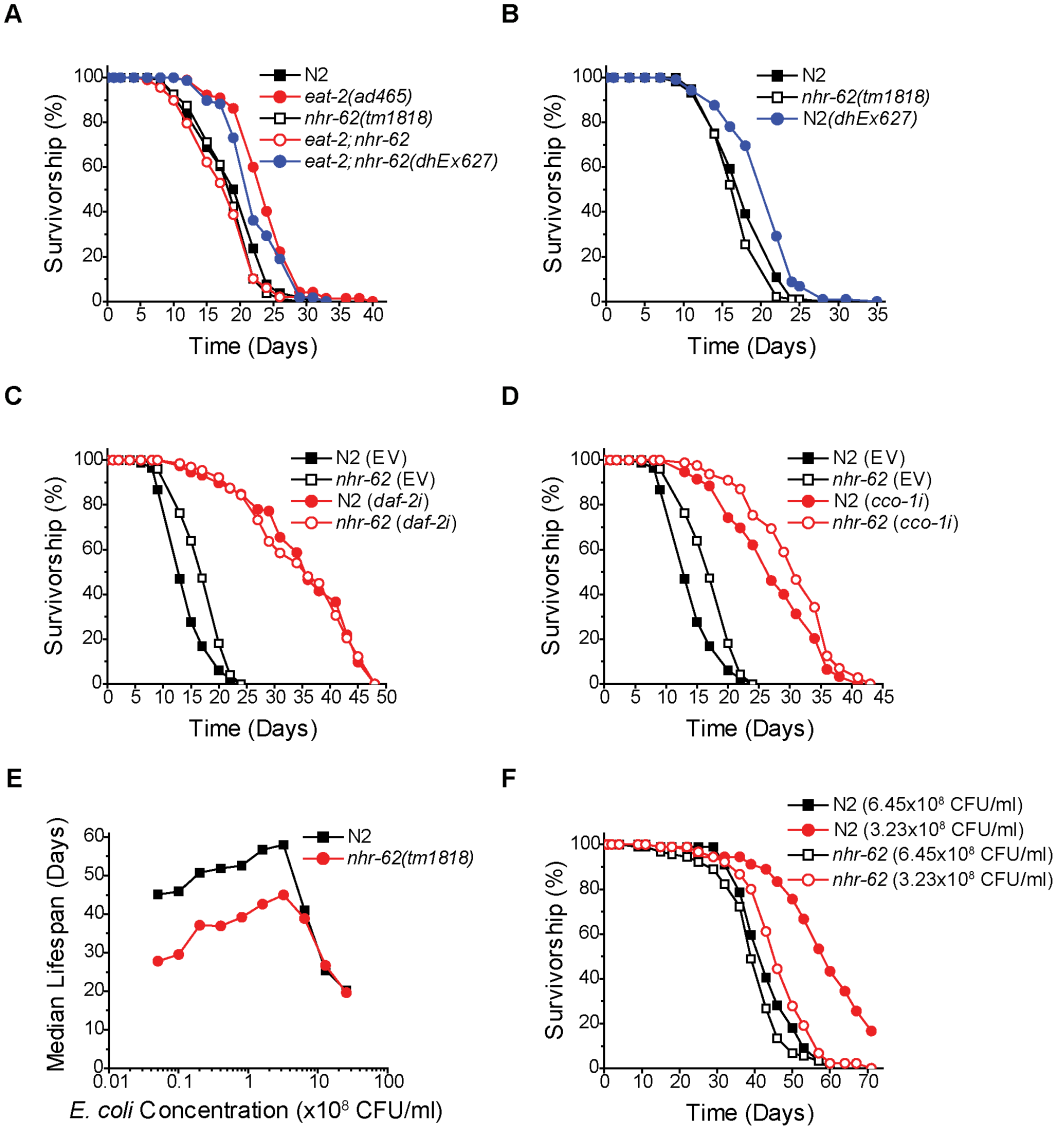
*nhr-62* specifically regulates DR-induced longevity. (A) Survivorship curves for wild-type (N2), *eat-2(ad465)*, *nhr-62(tm1818)*, *eat-2;nhr-62*, and *eat-2;nhr-62(dhEx627)*. Extra chromosomal array *dhEx627* (carrying a wild-type *nhr-62*) in *eat-2;nhr-62* resulted in a significant increase in lifespan compared to both *nhr-62(tm1818)* and *eat-2;nhr-62* mutants (p<0.001). (B) Wild-type (N2) worms with *dhEx627* exhibited a significant increase in lifespan compared to wild-type (p<0.001). See Figure S2 for physiologic traits of overexpression. (C,D) Both wild-type (N2) and *nhr-62(tm1818)* worms fed *daf-2* RNAi or *cco-1* RNAi had a significant increase in lifespan when compared to controls (p<0.001). (E) BDR curve for wild-type (N2) and *nhr-62(tm1818)* worms. The lifespan of wild-type and *nhr-62(tm1818)* were not different at the three most concentrated food dilutions, but were significantly different at the remaining 7 dilutions (p<0.001). (F) Survivorship curves for wild-type (N2) and *nhr-62(tm1818)* fed either 6.45×10^8^ CFU/ml or 3.23×10^8^ CFU/ml. p-values calculated by the log-rank test.

To confirm the role of *nhr-62* in DR-induced longevity, we generated an extra chromosomal line, *dhEx627*, expressing wild-type *nhr-62*, and introduced it into *eat-2;nhr-62* mutant animals to measure whether complementing *nhr-62* function was sufficient to restore longevity. As expected, the *dhEx627* array restored longevity to *eat-2;nhr-62* double mutants (Figure 2A). Interestingly, when this array was crossed into the wild-type background, *nhr-62* over-expressing worms displayed phenotypes similar to DR worms (*e.g.*, small body size and reduced fat) without affecting pharyngeal pumping rate (Figure S2). Furthermore, the *dhEx627* array significantly extended the life span of wild-type animals in four of eight independent experiments, suggesting that *nhr-62* can be sufficient to promote longevity (Figure 2B and Table S1).

Reduced insulin/IGF receptor (IR) signaling or reduced mitochondrial function have both been shown to extend life span [28], [29]. To test if *nhr-62* also modulates longevity of these pathways, we measured the lifespan of *nhr-62(tm1818)* animals fed *daf-2* (IR) or *cco-1* (cytochrome c oxidase) RNAi. As expected, *daf-2* RNAi and *cco-1* RNAi robustly increased the life span of wild-type animals. Moreover, RNAi knockdown similarly increased longevity in the *nhr-62(tm1818)* background (Figure 2C,D). These results reveal the *nhr-62* mutation does not affect these pathways, but specifically modulates DR-induced longevity. Taken together, these data indicate that NHR-62 is a novel regulator of DR-induced longevity.

### NHR-62 Mediates BDR Longevity Response at Lower Nutrient Levels

If NHR-62 is a robust mediator of DR-induced longevity, then it would be predicted to suppress longevity in a second DR regimen. To test whether *nhr-62(tm1818)* is also required for DR-induced longevity by BDR, we measured the lifespan of wild-type and *nhr-62(tm1818)* worms fed bacteria at ten different concentrations. We started with our *ad libitum* bacterial concentration of 2.5×10^9^ CFU/ml followed by nine subsequent two-fold serial dilutions. At the level of DR associated with the longest lifespan (optimal; 3.23×10^8^ CFU/ml), wild-type worms exhibited a 188% increase in median lifespan compared to *ad libitum* conditions (Figure 2E). When bacteria were diluted past this optimal DR condition, the median lifespan of wild-type worms decreased, resulting in a characteristic tent-shaped DR response curve. Unexpectedly, across the first two dilutions *nhr-62(tm1818)* worms exhibited a 40% increase in median lifespan compared to *ad libitum* conditions, identical to the wild-type DR response curve at these high nutrient conditions. However, at the optimal DR condition, the DR response curve for *nhr-62(tm1818)* mutants diverged from the wild-type curve; these animals were unable to respond to DR as effectively, with only a partial increase in the median lifespan at all subsequent concentrations (Figure 2E,F and Table S2). These results suggest that *nhr-62* blunts the BDR response at lower nutrient concentrations.

### NHR-62 Is Widely Expressed

**Figure 3:**
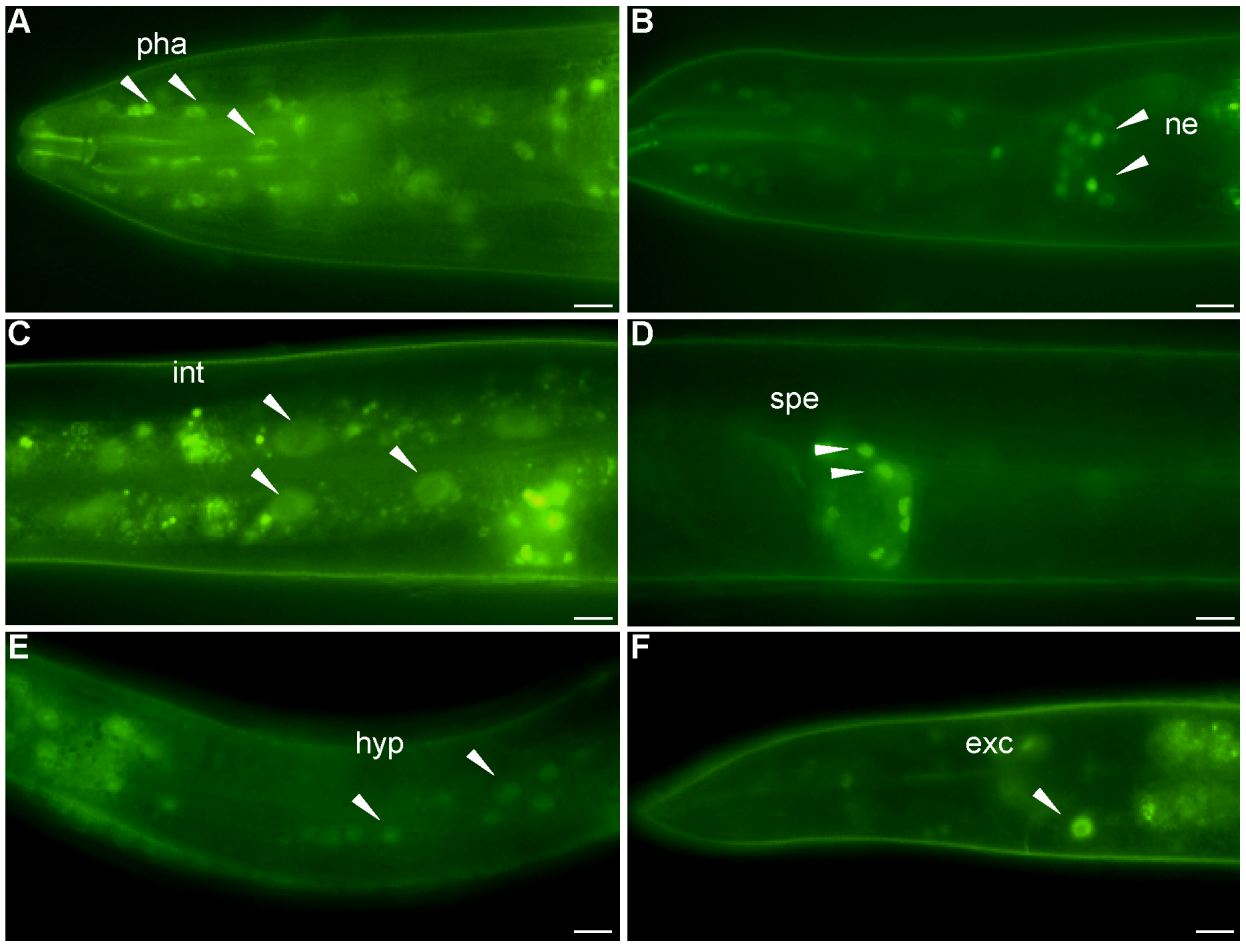
Expression pattern of *nhr-62*::*gfp.* A full length fusion of *nhr-62::gfp* resides in the nuclei of (A) pharyngeal cells (pha), (B) sensory neurons (ne) (C) intestinal cells (int) (D) spermatheca (spe) (E) hypodermis (hyp) (F) excretory cell (exc). Scale bar is 10 µM.

We obtained a transgenic strain from the Hope laboratory containing 2 kb of the *nhr-62* promoter fused to *gfp* (p*nhr-62*::*gfp*). As previously described, transgene expression was observed from embryo to adult in the pharynx and intestine. We also observed clear expression in various neurons including sensory neurons, motor neurons, hermaphrodite specific neuron, and pharyngeal neurons (data not shown). A similar pattern of expression was seen in the *eat-2(ad465)* background. A full-length integrated low copy *nhr-62::gfp* expressing strain from the TransgeneOme Project [30] was weakly expressed in many tissues including the nuclei of pharynx, sensory neurons, intestine, spermatheca, hypodermis, and excretory cell in both wild-type (Figure 3A–F) and *eat-2* backgrounds. Again no obvious change in *nhr-62::gfp* intensity or localization was observed in the *eat-2* background, nor were changes seen in *nhr-62* mRNA levels as measured by qPCR. Notably several tissues that express *nhr-62::gfp*, such as the intestine, ASI sensory neurons, and hypodermis, are major endocrine tissues that coordinate metabolic states and have been implicated in aging [10], [17], [20], [31], [32].

### NHR-62 Modulates DR-Induced Fat Metabolism

**Figure 4:**
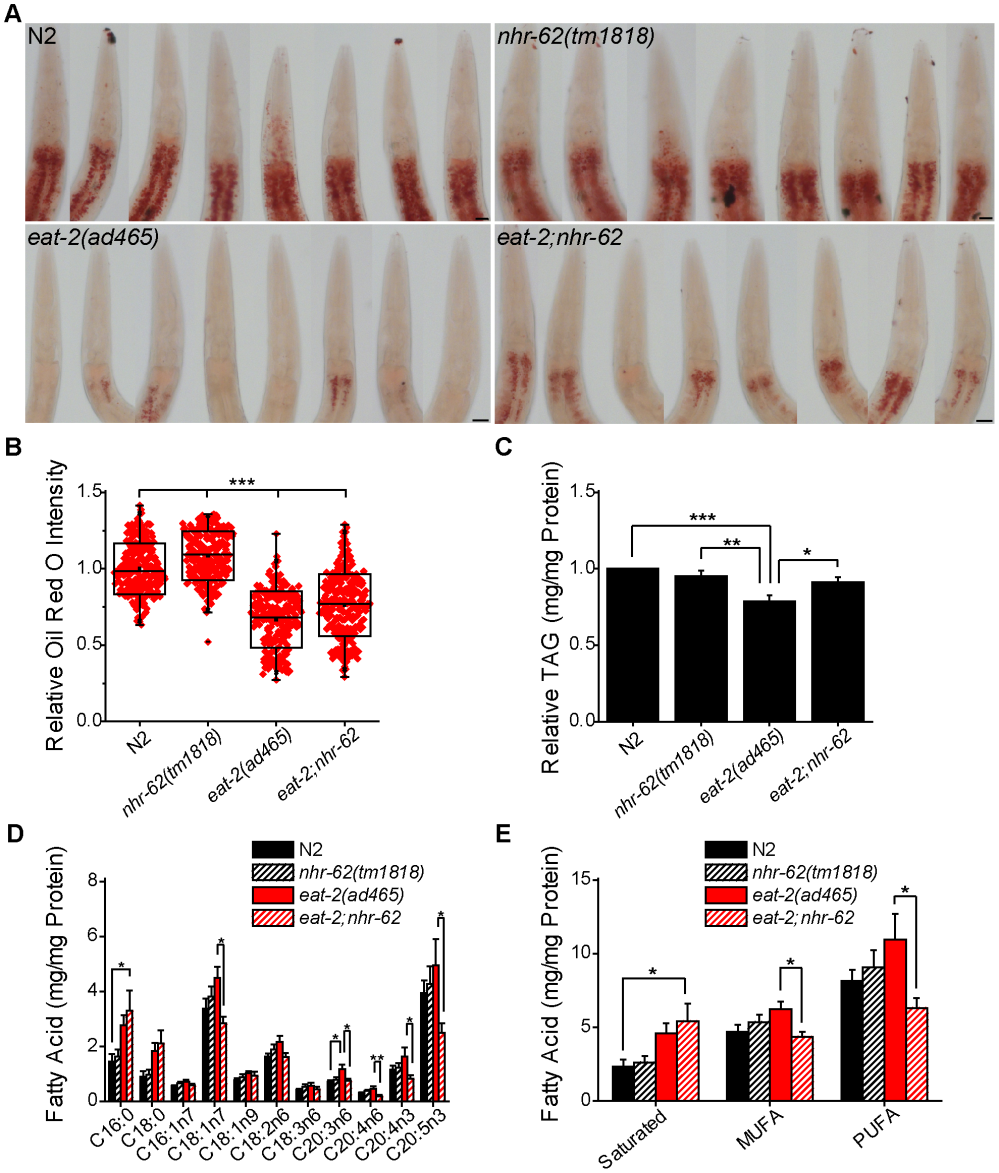
*nhr-62* modulates DR-induced fat metabolism. (A) Representative pictures of Oil Red O stained worms. Scale bar = 20 µM. (B) Oil Red O intensity is increased relative to wild-type (N2) or *eat-2(ad465)* controls by mutation of *nhr-62* (combined data from 3 independent experiments). (C) Total triglycerides of day 1 adults relative to wild-type (N2) (combined data from 16 independent experiments). (D) Fatty acid profiles of day 1 adult animals (combined data from 11 independent experiments). (E) Total saturated, monounsaturated fatty acid (MUFA), and polyunsaturated fatty acid (PUFA) calculated from (D). *eat-2;nhr-62* had increased saturated fat compared to wild-type (N2) and decreased MUFAs and PUFAs compared to *eat-2(ad465)*. See Figure S3 for physiologic traits of *nhr-62* mutation. *p<0.05, **p<0.01, ***p<0.001 by single factor ANOVA with Tukey test. Mean (Center Line) ± SD (Box) with bars representing an outlier coefficient of 1.5, or Mean±SEM.

Animals under DR generally display dramatic changes in stored fat compared to animals in *ad libitum* conditions. Since *nhr-62* shares homology to HNF4α-like NHRs known to function in fat metabolism such as *nhr-49* and *nhr-80* [33], [34], we hypothesized that *nhr-62* might be necessary for proper fat regulation under DR. To test this hypothesis, we treated wild-type, *nhr-62(tm1818)*, *eat-2(ad465)*, and *eat-2;nhr-62* animals with the lysochrome dye Oil Red O, which stains neutral triglycerides and lipids (Figure 4A). The intensity of Oil Red O has been reported to correlate positively with triglyceride levels as measured by biochemical assays [35]. We found Oil Red O staining intensity in *eat-2* mutant animals was noticeably less than wild-type. Notably, mutation of *nhr-62* modestly increased staining intensity relative to wild-type or *eat-2* controls. *eat-2;nhr-62* mutants had a moderate but significant 14.1% increase in staining intensity compared to *eat-2* animals (Figure 4B). This difference was not due to increased feeding as pumping rates of *eat-2* and *eat-2*;*nhr-62* were identical, as were body size and progeny production (Figure S3). We also measured the levels of triglycerides (TAGs) and similarly observed that *eat-2* had a significant decrease in TAG/protein compared to both wild-type and *nhr-62* mutants. Furthermore, *nhr-62* partially reversed this effect, giving a significant 15.6% increase in total TAG/protein in *eat-2*;*nhr-62* worms compared to *eat-2* worms (Figure 4C). These data indicate a general role for *nhr-62* in regulating lipid levels.

Longevity in *C. elegans* has been associated with altered fatty acid composition. For example, loss of germline stem cells, which increases life span, results in increased monounsaturated fatty acid content, and this longevity depends on production of oleic acid (C18:1n9) [36]. To determine if *nhr-62* regulates fatty acid composition under DR, we quantified individual fats by gas chromatography. *eat-2;nhr-62* double mutants exhibited increased levels of saturated fatty acids compared to wild-type. Moreover, *eat-2;nhr-62* double mutants showed a significant reduction of various monounsaturated fatty acids (MUFAs) and polyunsaturated fatty acids (PUFAs) compared to *eat-2* mutants alone (Figure 4D,E). These observations suggest that *nhr-62* modulates changes in fatty acid composition under DR. Interestingly, we observed that the mRNA level of *fat-2*, a key enzyme in the production of PUFAs, was up-regulated 1.6-fold in *eat-2* worms compared to wild-type as measured by qPCR. Mutation of *nhr-62* dampened this up-regulation, although this effect did not reach statistical significance (Figure S4), suggesting that post-transcriptional regulation might also be at work.

**Figure 5:**
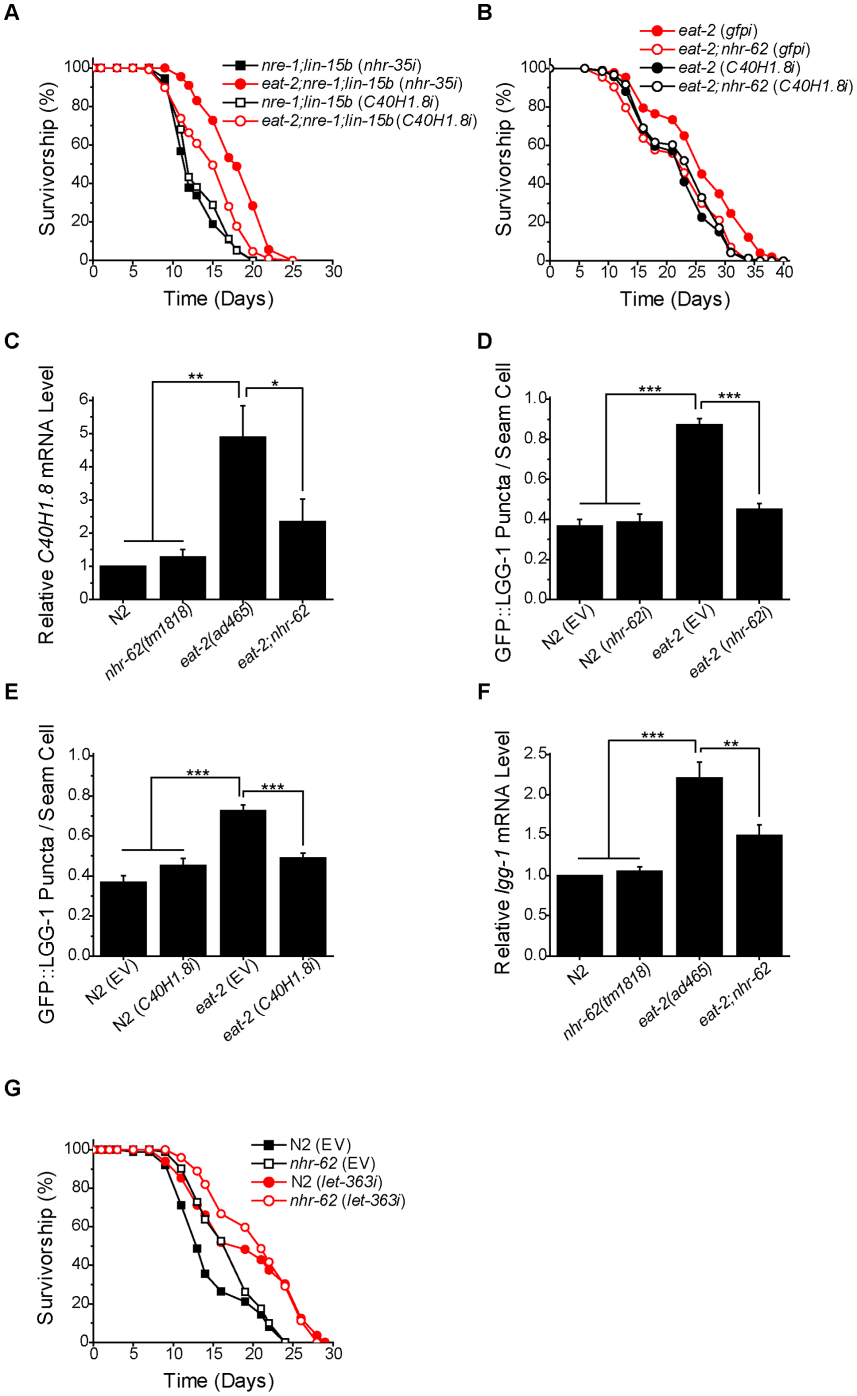
*nhr-62* regulates a DR-induced lipase and autophagy. (A) *eat-2;nre-1;lin-15b* worms fed *C40H1.8* RNAi had a significantly reduced lifespan compared to *eat-2;nre-1;lin-15b* worms fed *nhr-35* control RNAi (log-rank test p<0.001). See Figure S5 for additional experiments. (B) *C40H1.8* RNAi did not decrease the lifespan of *eat-2;nhr-62* worms compared to *eat-2;nhr-62* (*gfpi*). (log-rank test p = 0.4798). (C) The up-regulation of *C40H1.8* expression under DR was significantly suppressed in *eat-2;nhr-62*. (D) The induction of GFP::LGG-1 puncta formation in *eat-2(ad465)* was significantly suppressed to *ad libitum* levels by *nhr-62* RNAi (combined data from 3 independent experiments). (E) The induction of GFP::LGG-1 puncta formation in *eat-2(ad465)* was significantly suppressed to *ad libitum* levels on *C40H1.8* RNAi (combined data from 3 independent experiments). (F) The up-regulation of *lgg-1* expression under DR was significantly suppressed in *eat-2;nhr-62*. (G) Both wild-type (N2) and *nhr-62(tm1818)* worms fed *let-363*/TOR RNAi had a significant increase in lifespan when compared to controls (log-rank test p<0.001). EV = empty vector . *p<0.05, **p<0.01, ***p<0.001 by single factor ANOVA with Tukey test. Mean±SEM.

Lipases are involved in the liberation of fatty acids from triglyceride stores. Given the changes in fatty acid composition and previous studies implicating lipases in modulating longevity [37], [38], we hypothesized that lipases might modulate DR-induced longevity. To test this hypothesis we screened through 34 predicted lipases in *C. elegans* for suppression of longevity in the *eat-2;nre-1;lin-15b* background (Table S3). Interestingly, we found that RNAi targeting one predicted lipase, *C40H1.8*, partially reduced longevity of dietary restricted animals, reaching significance in 4 out of 6 experiments (Figure 5A and Figure S5, Table S1). One explanation for a partial response is that *C40H1.8* RNAi does not result in complete knockdown (Figure S6). Additionally, *C40H1.8* has two closely related homologs, *C40H1.7* and *C40H1.9*, which were unaffected by *C40H1.8* RNAi knockdown and might compensate for its reduction of function (Figure S6). Consistent with a unified pathway, *C40H1.8* knockdown did not further reduce the life span of *eat-2;nhr-62* double mutants (Figure 5B). By qPCR analysis, we found that *C40H1.8* was up-regulated five-fold under DR. In *eat-2*;*nhr-62* animals, this up-regulation was attenuated by approximately 50%, suggesting some regulation of *C40H1.8* expression by *nhr-62* (Figure 5C). These data indicate that *C40H1.8* may play at least a partial role in mediating *eat-2*-induced longevity. *C40H1.8* contains a class 3 lipase domain, found in a variety of proteins predicted to have triacylglycerol lipase activity. In particular, *C40H1.8* contains the catalytic triad comprised of serine, aspartate, and histidine (S198, D256, H316), a nucleophilic elbow, and other features typical of lipases (Figure S7) [39], [40]. Although C40H1.8 does not have a clear mammalian ortholog, class 3 lipases from other species include Atg15p, which is involved in autophagy in *Saccharomyces cerevisiae* and is required for DR-mediated longevity [38], and the Sn-1 specific diacylglycerol lipase implicated in endocannabinoid signaling in mice [41].

### NHR-62 Regulates DR-Induced Autophagy

Autophagy, the catabolic process involving the degradation of cellular components which are recycled to serve as a source of energy and precursors for biosynthesis, has also been linked to DR, as well as to fatty acid metabolism and lipolysis [20], [42], [43]. To determine if *nhr-62* influenced DR induced autophagy, we utilized a GFP::LGG-1 reporter strain. LGG-1 is the worm ortholog of *S. cerevisiae* ATG8p, which is incorporated into pre-autophagosomal membranes, and is necessary for proper cellular degradation during autophagy [44]. Under normal conditions GFP::LGG-1 is cytoplasmic and diffuse in the worm's hypodermal seam cells, but under DR conditions, forms puncta which are associated with an increase in autophagy [20]. We observed a significant increase in the average number of GFP::LGG-1 puncta per seam cell in the *eat-2* background, and found that *nhr-62* RNAi suppressed puncta formation back to wild-type levels, suggesting that *nhr-62* modulates autophagy up-regulation under DR (Figure 5D). Previous studies have shown that lipases are required for autophagy induction in the context of the gonadal longevity pathway [43]. We wondered whether the *C40H1.8* lipase implicated here in *eat-2* longevity played any role in autophagy, and found that *C40H1.8* RNAi knockdown reduced the number of GFP::LGG1 puncta in the *eat-2* background (Figure 5E). Additionally, levels of *lgg-1* mRNA were likewise induced in *eat-2* mutants in an *nhr-62* dependent manner (Figure 5F). These results suggest that *nhr-62* regulates DR-mediated longevity, possibly through lipolysis and autophagy.

A major regulator of the DR response is the target of rapamycin (TOR) kinase. Down-regulation of *let-363*/TOR induces autophagy and extends life span through DR pathways [21]. To see how *nhr-62* interacts with TOR signaling, we reduced *let-363*/TOR by RNAi in the *nhr-62(tm1818)* mutants. We found that *let-363* knockdown induced longevity in both wild-type and *nhr-62* mutants (Figure 5G and Table S1). These results suggest that *let-363*/TOR acts downstream or parallel to *nhr-62*.

### NHR-62 Regulates the DR-specific Transcriptome

Because *nhr-62* promotes DR longevity and associated physiological processes, we speculated that it could modulate the overall transcriptional change upon DR. To test this hypothesis, we performed RNA-seq on wild-type, *nhr-62(tm1818)*, *eat-2(ad465)*, and *eat-2;nhr-62* animals. This approach not only enabled us to identify genes regulated under DR, but also which genes showed *nhr-62* dependence. Our criteria for differential gene regulation included a greater than 1.5-fold change in expression compared to wild-type, a false discovery rate less than 0.05, and greater than 10 reads when normalized to the base mean. For each sample we typically obtained 25–37 million unambiguous reads, and could assign these to approximately 15,000 genes. Heat maps of the mean Euclidean distance between samples showed that *nhr-62* was most similar to wild-type, while *eat-2* was most different, with *eat-2;nhr-62* in between (Figure S8). qPCR on 12 of 13 regulated genes showed consistency with RNA-seq data, validating this approach for our analysis (Figure S8).

**Figure 6:**
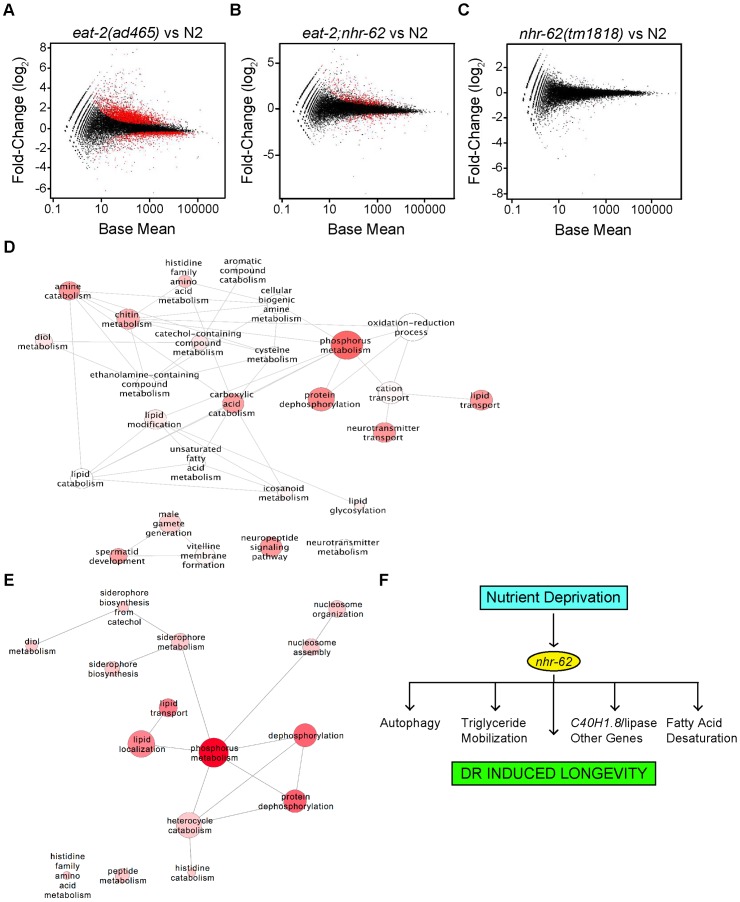
Expression profile analysis of *eat-2* and *nhr-62* regulated genes. Scatter plots of log2 fold-changes versus mean expression level. Red dots highlight differentially expressed genes at FDR<0.05 for (A) *eat-2(ad465)* versus wild-type (N2) (B) *eat-2;nhr-62* versus wild-type (N2) and (C) *nhr-62(tm1818)* versus wild-type (N2). (D) ReviGO analysis to summarize all GO-BP terms returned by DAVID with p value <0.1 when analyzing Gene-list “*eat-2*-vs-N2” and (E) list of DR regulated genes rescued by *nhr-62(tm1818)*. Bubble size indicates the frequency of the GO term from the GOA database, bubble color is dictated by the p value with a darker color being a lower p value, and similar GO terms are linked by lines where the line thickness indicates degree of similarity. (F) Model for DR. See Figure S8 for Euclidean distances between samples, Venn diagram, and qPCR validation of RNA-seq data.

Global profiles comparing wild-type and *eat-2* gave rise to over 3,000 genes with significant gene expression changes out of 17,788 examined genes, depicting an altered transcriptome for longevity induced by the *eat-2* mutation (Figure 6A and Table S4). DAVID analysis [45], [46] revealed an enrichment of biological processes involved in oxidation/reduction, phosphorus metabolism, unsaturated fatty acid, eicosanoid and ceramide metabolism, lipid modification and transport, amino acid, amine and chitin metabolism, and neuropeptide signaling among others (Figure 6D and Table S5).

Consistent with a physiological function in mediating DR, *nhr-62* mutation restored expression of over 600 genes regulated by *eat-2(ad465)* back to wild-type levels (Figure 6B and Table S6). By contrast, little change was seen in the *nhr-62* transcriptome compared to wild-type (Figure 6C), with only 17 genes differentially expressed. Enriched categories regulated by *nhr-62* under DR included genes involved in fatty acid localization and transport (*vit-1/2/3/4/5/6*, *lbp-8*, ABCG transporters, *wht-5* and *wht-9*), phosphorus metabolism (kinases *gska-3* and *T21G5.1*, phosphatases *Y57G11C.6* and *B0280.11*), nucleosome assembly and organization, (*C50F4.6*, *ZC155.2*) protein, amino acid, histidine, and heterocyclic amine metabolism (*zmp-1*, *T25B9.6*, *B0019.1*) and short chain dehydrogenase metabolism (*dhs-25*, *dhs-14*) (Figure 6E and Tables S6,S7). Other genes up-regulated by *eat-2* but reversed by *nhr-62* mutation include the glutathione S-transferase *gst-21*, collagen *col-146*, and the FMRFamide-related neuropeptide *flp-21.* Genes down-regulated under DR and reversed by *nhr-62* include the acyl CoA dehydrogenase, *acdh-2*, lipid binding protein *lbp-8*, the conserved transmembrane protein *C09B8.4*, and the vitellogenins *vit-1-6*. The overall changes in transcription seen in *nhr-62* mutants under DR, supports the notion that *nhr-62* is an important mediator of the DR response.

## Discussion

Nuclear hormone receptors are critical regulators of animal metabolism, development and homeostasis, and are particularly well suited to couple nutrient and lipid availability to transcriptional cascades. By screening through the set of NHR genes in *C. elegans* we identified the HNF4α homolog, *nhr-62*, as an important mediator of the DR response. Several lines of evidence are consistent with the notion that *nhr-62* promotes aspects of DR-induced metabolism and longevity. RNAi knockdown or mutation of *nhr-62* specifically suppressed *eat-2* DR-induced longevity, but not that of reduced insulin/IGF receptor (*daf-2*) or mitochondrial function (*cco-1*) arguing for a DR specific function. Importantly, *nhr-62* mutation prevented animals from fully responding to the longevity-inducing effects of bacterial food dilution (BDR), thereby making it one of only a handful of DR-regulating genes that clearly modulates multiple forms of DR. By comparing *eat-2* to *eat-2;nhr-62* mutants we found a reversal of Oil Red O staining intensity, triglyceride levels, and autophagy induction, without seeing a difference in body size or pumping rate. Additionally, we found that *nhr-62* mutation decreased the mono- and polyunsaturated fatty acid content of dietary restricted worms. Finally, we found a reversal of the DR-induced transcriptional regulation of a predicted lipase required for DR, as well as a plethora of genes through RNA-seq analysis. These results point to a model in which a dietary restricted state promotes activation of *nhr-62*, the lipase *C40H1.8*, and numerous other genes to modulate DR-induced longevity, possibly through fat metabolism, autophagy, and other processes (Figure 6F). Although *nhr-62* was the most visible candidate that emerged from our screens, it is possible that other NHRs may also play a role in the DR response, and were missed due to either incomplete knockdown or functional redundancy.

It was unexpected to see a partial BDR response by *nhr-62(tm1818)* mutants. One potential explanation for this observation is that by mutating *nhr-62*, the optimum DR threshold has been shifted to a new concentration. However, *nhr-62(tm1818)* mutants do not exhibit a globally shifted BDR response curve since the highest three food concentrations give median life spans that align precisely with wild-type. At further food dilution, *nhr-62(tm1818)* mutation significantly suppressed life span extension compared to wild-type, which results in a parallel response but with lower median life spans compared to wild-type. These results are consistent with a blunted response to DR in the lower nutrient range. In contrast to a “master regulator” of DR, which might completely abrogate life span extension at all food concentrations, this type of response curve could be indicative of a gene that plays a role in modulating or fine tuning the BDR longevity response at specific nutrient levels. It is also possible that *nhr-62* mutation disrupts metabolism in a non-specific manner that is incompatible with a proper DR response. What argues against this is that *nhr-62* mutation on its own had very little effect on gene expression or physiology under *ad libitum* conditions, suggesting *nhr-62* is engaged in a regulatory role primarily under DR states.

*nhr-62* exhibits several phenotypes suggesting it functions in mobilizing fat to partition energetic and biosynthetic demands under nutrient limitation. Notably *nhr-62* mutation prevents some of the reduction of TAG and Oil Red O staining stores seen under DR longevity. Several dynamic changes in fatty acid metabolism have been observed upon DR shifts in other species. In flies there is a shift toward increasing fatty acid synthesis and breakdown, and inhibiting this suppresses DR longevity [47]. Mice initially increase endogenous fatty acid synthesis followed by prolonged fatty acid oxidation [48]. We observed that deletion of *nhr-62* alters fatty acid composition in the *eat-2* background, increasing the amount of the saturated fatty acid palmitate, and reducing the amount of various MUFAs and PUFAs, including C18:1n7, C20:3n6, C20:4n6, C20:4n3, and C20:5n3 fatty acids. It is possible that these changes could contribute towards the suppression of life span. Interestingly, a handful of these fatty acids have been recently implicated in longevity. The omega-6 fatty acid, DGLA (C20:3n6) stimulates autophagy and extends *C. elegans* life span [49]. Oleic acid (C18;1n7) has been shown to be important for life span extension upon germline removal [36]. It seems plausible that some of these lipids or their derivatives function in DR-mediated longevity. Additionally germline removal also results in up-regulation of the lipase, *lipl-4*, which stimulates autophagy [43]. Our results suggest a model in which lipolysis by *C40H1.8* lipase could stimulate autophagy, since RNAi against this gene diminished DR longevity and GFP::LGG-1 punctual formation. Alternately, lipolysis could liberate fatty acids that serve a signaling role, binding to NHR-62 or related receptors. Future studies should further clarify the complex relationships between fatty acid composition, lipolysis, autophagy and longevity in the DR response.

By RNA-seq analysis we identified significant changes in the expression of approximately 3,000 genes comparing *eat-2* to wild-type. Amongst enriched categories are genes involved in phosphorus metabolism, unsaturated fatty acid metabolism, eicosanoid and ceramide metabolism, lipid modification and transport, amino acid, amine and chitin metabolism, and neuropeptide signaling. Notably mutation of *nhr-62* reversed the regulation of about 600 of these genes, suggesting a key role in mediating the DR response. These genes are candidates for *nhr-62* targets, and possibly important effectors of the DR response (Table S6). Among them are genes whose molecular identity suggests a role in fat metabolism, including transport (apolipoproteins/vitellogenins, *vit-1* through *vit-6*, ABCG transporters *wht-5*, *wht-9*), lipid binding (*lbp-7*, *lbp-8*), fatty acid remodeling (acyl coA thiolases *F57F4.1*), β-oxidation (*acdh-2*, *ech-7*, acyl transferase *acl-13*) and fatty acid elongation (*elo-7*, *elo-8*). Presumably some of these genes may mediate the physiologic changes relevant to DR. Indeed, vitellogenin knockdown has been previously shown to extend life [50].

Dietary restriction is associated with reduced TOR signaling, reduced protein synthesis, and increased autophagy [20], [21], [51]. Within the TOR pathway, the amino acid sensing G-proteins RAGA-1, RAGC-1 and TOR itself were down regulated about 20% but was significant for TOR only. Consistent with a possible downstream or parallel role, *let-363/*TOR RNAi induced longevity in the *nhr-62* mutant background. We also found that the autophagy gene *lgg-1* was up-regulated 1.5-fold, in a manner dependent on *nhr-62*. Consistently, we observed that autophagic vesicles visualized with GFP::LGG-1 were increased under DR in an *nhr-62* dependent manner. Previous studies have shown that *lgg-1* and other autophagy genes are required for DR and various longevity pathways [52]. However, other autophagy genes did not show significant regulation under DR, suggesting that post-transcriptional mechanisms contribute to this response.

Reduced protein translation extends life span and may be one of the important outputs of the DR response [53]. We observed significant down regulation of several translation initiation and release factors including *inf-1*, whose down-regulation has been shown to extend life span [54]. Additionally, a systematic, albeit small (approximately 20%) down-regulation of scores of ribosomal proteins was evident under DR; however none of these showed definitive *nhr-62* dependence. It is possible that these minor transcriptional changes collectively recapitulate the physiologic effect of DR. Alternately, modest regulation of a critical enzyme or key regulator in the pathway might be sufficient. *nhr-62* might also regulate unidentified post-transcriptional modifiers of these processes, especially given the many kinases and phosphatases affected (Tables S6,S7). Many of the genes regulated by *nhr-62* also include nonconserved genes that fall into large families including F-box proteins, lectins, activated in blocked unfolded protein response (abu) and fungal induced proteins, which are speculated to be involved in innate immunity. In the future it will be important to understand how the diverse transcriptional output of *nhr-62* relates to longevity and to identify direct targets by ChIP-seq.

It is currently unclear which mammalian homolog is most functionally analogous to *nhr-62*. The *C. elegans* genome has undergone a radical expansion of the HNF4 family of nuclear receptors, making an assignment of homologous function for *nhr-62* challenging. One such HNF4-like nematode receptor, *nhr-49*, has been proposed as PPARα-like because it is involved in the starvation response, turns on genes implicated in β-oxidation, fatty acid desaturation, binding and transport. However, in our hands, *nhr-49* knockdown did not abrogate the DR response. Conceivably, *nhr-62* functions similar to the PPARs as well, since these nuclear receptors are known lipid sensors that regulate metabolism under different nutritional states. Corton *et al* originally observed a substantial overlap in the expression profile of DR and PPAR nuclear receptor regulation in mice [55]. Furthermore, in non-human primates, regulation of PPARs may be pivotal for the effects of DR [56]. Similar meta-analysis of expression profiles implicates PPARα in the DR response [57]. It is also possible that *nhr-62* functions like HNF4, which regulates apolipoprotein levels and lipid and glucose homeostasis. Notably, both PPARs and HNF4 nuclear receptors can bind to fatty acids, which may be regulating their activity [58], [59]. The finding that *nhr-62* is involved in fat metabolism raises the possibility it too is regulated by fatty acid like ligands.

Interestingly, the *C. elegans* transcription factor SKN-1/NF-E2 regulates DR-induced longevity from the pair of ASI sensory neurons [17]. This implies that cell non-autonomous signals downstream of SKN-1/NF-E2 mediate a systemic physiological response to DR. Conceivably, this DR response could be communicated through an endocrine mechanism, and suggest that specific hormones and hormone receptors such as NHR-62 are required for DR-induced longevity. If so, it would be exciting to identify the ligand for NHR-62 and determine if it could promote longevity under replete conditions. Alternately, NHR-62 may not be ligand regulated, but instead function as a competency factor instructed by other regulatory molecules that control its response to nutrient availability and facilitate metabolic remodeling. In the future it will be interesting to distinguish these possibilities. To our knowledge, this study is the first to find a nuclear hormone receptor specifically required for DR-induced longevity, potentially though regulation of fat metabolism and autophagy.

## Materials and Methods

### *C. elegans* Strains

All strains were grown and maintained on NGM agar seeded with *E. coli* (OP50) at 20°C unless otherwise noted. Standard procedures for culturing and maintaining strains were used [60]. Strains used: N2 (wild-type), *eat-2(ad465)*, *nhr-62(tm1818)*, *nre-1(hd20);lin-15b(hd126)*, UL1385 mvEx5591*(Y67A6A.2*::*gfp* + *unc-119)*, *eat-2(ad465);*mvEx5591*(Y67A6A.2*::*gfp* + *unc-119)*, OP403 wgIs403*(nhr-62*::TY1 EGFP 3× FLAG (91H02);*unc-119(+))*; *unc-119(tm4063)*, QU1 izEx1*(*p*lgg-1*::*gfp*::*lgg-1* + *rol-6)*, *eat-2(ad465)*; izEx1(p*lgg-1*::*gfp*::*lgg-1* + *rol-6*), *eat-2(ad465);nhr-62(tm1818)* dhEx627(p*myo-3*::*cfp* + fosmid WRM065CF04). The dhEx627 extra-chromosomal rescue strain was generated by injecting DNA from worm fosmid WRM065CF04 (10 ng/µl), a co-injectable marker (p*myo-3*::*cfp* at 20 ng/µl) and salmon sperm DNA at 70 ng/µl. Stable arrays were selected for and maintained based on expression of the co-injected marker. For imaging of *nhr-62::gfp*, day 1 adult worms were visualized using a Zeiss Axioskop2 Plus microscope and representative images were processed with ImageJ [61].

### Lifespan Assays

Lifespans were recorded as previously described [62] unless otherwise noted. A log-rank p value of less than 0.001 was used for establishing significance. Experimental worms were grown at 20°C for three generations without starving and were staged as eggs on experimental plates using a timed egg lay. L4s were moved into the appropriate conditions for the start of the aging experiment at a density of 10–12 worms per 6 cm plate. At the L4 stage, or day 0 of aging experiment, worms were moved to fresh experimental plates at density of 10–12 worms per plate. Worms were scored every other day across their lifespan for movement. Worms that did not move on their own were gently touched with a sterilized platinum wire pick and monitored for movement. If there was no movement worms were scored as dead. During these experiments worms were transferred to fresh experimental plates every other day until the end of the reproductive period and then moved once a week. Animals that crawled off the plate, exploded due to a ruptured vulva, or became Egl (egg laying defective), in which unlaid eggs hatch inside the mothers, were censored from the aging experiment. The number of aging experiments performed, mean, median, and maximum lifespans, log-rank analysis, number of worms used in each experiment and worms censored were recorded (Table S1). For the high-throughput aging screen, worms were grown to gravid adult and bleached to collect staged eggs. To increase the efficacy of RNAi, RNAi sensitive strain *nre-1(hd20);lin-15b(hd126)* was used. Eggs were pipetted at a target density of 15 eggs per well into 12 well cell culture plates with RNAi NGM seeded with RNAi clone of interest. At L4, or day 0 of adult, 15 µl of FUdR (5-Fluoro-2′-deoxyuridine) (50 mM) was pipetted into each well to inhibit progeny production. On day 7 of adulthood, the number of worms alive was determined for each well. On day 15 of adulthood, the number of worms alive was again determined and the percent surviving was calculated. For RNAi experiments, worms were maintained on NGM plates with 20 µg/ml carbenicillin, 10 µg/ml tetracycline, and 1 mM IPTG. Worms were transferred to fresh RNAi plates every other day. For BDR longevity experiments, lifespans were recorded as previously described [10]. Worms were grown for three generations without starving on NGM plates seeded with OP50 and then staged using a timed egg lay. At L4, or day 0 of aging experiment, worms were transferred to NGM plates with FUdR (100 µg/mL). After 48 hours, worms were then washed for one hour in BDR liquid media (5.85 g NaCl, 1 g K_2_HPO_4_, 6 g KH_2_PO_4_, 1 ml cholesterol at 5 mg/mL in ethanol, and MilliQ H_2_O to 1 L) with carbenicillin (50 µg/ml), kanamycin (10 µg/ml), and tetracycline (1 µg/ml) added to inhibit bacteria replication. 90 worms were then moved into defined bacteria concentrations and scored every 3–4 days for survival at a density of 15 worms per well. On every score day worms were transferred to freshly made bacteria condition. FUdR was added to the BDR liquid media for the first 14 days of adulthood to inhibit progeny production. Bacteria concentration was determined through serial dilution, plating, and counting of colony forming units (CFUs). The number of aging experiments performed, mean, median, and maximum lifespans, log-rank analysis, number of worms used in each experiment and worms censored were recorded (Table S2).

### Fat Analysis

Oil Red O protocol was adapted from [35]. 200–300 day 1 adult worms synchronized through a timed egg lay were washed with M9 and fixed with 50% isopropanol for 15 minutes. Oil Red O stock solution (0.5 g Oil Red O in 100 ml isopropanol) was diluted in water to 60% Oil Red O working solution and used to stain the worms overnight. Worms were then washed with PBS and 0.01% TritonX-100 (diluted in PBS) was added prior to mounting the worms. Images were captured using a DIC microscope and Zeiss AxioCam MRc5 camera. ImageJ was used to invert the color images and a 100 pixel diameter circle was drawn to quantify the intensity of the first intestinal cell and background was subtracted out. Statistics were done using GraphPad Prism software.

Biochemical triglyceride assays were performed using approximately 5,000 day 1 adult worms staged through timed egg lay. Worms were from NGM plates and washed 3 times in PBS then ground up using a pestle and homogenized using a Branson Sonifier Cell Disruptor. Triglyceride and protein levels were determined using a Triglyceride Colorimetric Assay Kit (Cayman Chemical Company) and the Pierce BCA Protein Assay Kit (Thermo Scientific) and analyzed on a Biotek Power Wave XS plate reader according to manufacturer's instructions. Three technical replicates were preformed for every biological replicate. Standards were also done in triplicate. Statistics were done using GraphPad Prism software.

### GC Analysis of FAMEs

Day 1 adults animals were collected in 100 µl M9 buffer, washed 2-times, and run through 10-freeze/thaw cycles before being sonicated to lyse the cuticle and tissue. Protein concentration was determined using the Pierce BCA Protein Assay Kit (Thermo Scientific) according to manufacturer's instructions. 50 µl was removed to a 1.5 ml gas chromatography (GC) vial for derivatization. To each vial, 200 ng each of methyl C11:0, C13:0, and C23:0 (NuChek Prep, Inc.; 20 µg/ml stock in methanol) were added as internal reference standards. To convert fatty acids into their fatty acid methyl ester (FAME) derivatives, 0.5 ml of 2.5% H_2_SO_4_ (Sigma) in methanol (Biosolve) was added to each sample and incubated at 80°C for 20 minutes with shaking. 0.75 ml of water (Biosolve, ULC Grade) and 350 µl of hexane (Sigma) were next added and the sample was incubated with shaking for 10 min to extract FAMEs into the hexane layer. The hexane layer was removed, concentrated by evaporation, and used directly for analysis. FAME analysis was carried out using an Agilent 7890A GC equipped with a flame ionization detector (FID) (Agilent Technologies, Inc) and a DB-23 column (30 m×0.25 mm I.D., 0.25 µm, Agilent) using helium as a carrier gas at a flow rate of 3 ml/min. 1 µl per sample was injected in pulsed-splitless mode. The initial oven temperature was set to 80°C, held for 1 minute; increased to 170°C at 6.5°C/min; then increased to 215°C at 2.75°C/min. FAMEs were identified and compared against known reference standards for quantification.

### qRT-PCR

Synchronized worms were collected into TRIzol (Invitrogen) when reaching adulthood but bearing no embryos. Total RNA and cDNA was prepared using RNeasy Mini kit (QIAGEN) and iScript cDNA Synthesis Kit (Bio-Rad) respectively. qRT-PCR was performed with Power SYBR Green master mix (Applied Biosystems) on a ViiA7 384 Real-Time PCR System (Applied Biosystems). A combination of *ama-1* and *cdc-42* was used as control. 4 biological replicates containing 300–400 worms each and four technical replicates were tested for each experiment. qPCR primers sequences are in Table S8.

### Autophagy Marker GFP::LGG-1

Counting of puncta formation was done as previously described [20]. Briefly, young adults were transferred to RNAi plates targeting the gene of interest. L3 animals of the next generation were scored. The number of GFP puntca in each seam cell was recorded using a Zeiss Axioskop2 Plus microscope. For *nhr-62* RNAi experiments, 3 biological replicates with 31 animals (345 seam cells) of GFP::LGG-1 (L4440), 26 animals (225 seams cells) of GFP::LGG-1 (*nhr-62i*), 62 animals (773 seams cells) of *eat-2* GFP::LGG-1 (L4440), and 58 animals (598 seam cells) of *eat-2* GFP::LGG-1 (*nhr-62i*) were scored. For *C40H1.8* RNAi experiments, 3 biological replicates with 50 animals (323 seam cells) of GFP::LGG-1 (L4440), 43 animals (382 seams cells) of GFP::LGG-1 (*C40H1.8i*), 100 animals (933 seams cells) of *eat-2* GFP::LGG-1 (L4440), and 99 animals (896 seam cells) of *eat-2* GFP::LGG-1 (*C40H1.8i*) were scored.

### RNA-seq and Bioinformatic Analysis

Worms of indicated genotypes were synchronized by two rounds of bleaching and approximately 300 worms for each genotype were handpicked into TRIzol (Invitrogen) when they just reached adulthood without carrying embryos. Total RNA was prepared with RNeasy Mini Kit (QIAGEN). cDNA library was subsequently constructed by TruSeq RNA Sample Preparation Kit (Illumina). Illumina sequencing (100 bp single end) was performed on 3 biological replicates for each of the 4 treatments *eat-2*, *nhr-62;eat-2*, *nhr-62*, N2, giving a total of 12 samples. Sequencing reads were clipped to remove adapter sequence using fastx_clipper (http://hannonlab.cshl.edu/fastx_toolkit/), clipped reads were aligned to the *C. elegans* reference genome (WBcel215.67) using tophat version 1.3 (Trapnell, Pachter & Salzberg 2009) with option −g1 to ensure unique mapping and option −G to pass the Caenorhabditis_elegans.WBcel215.67.gtf. The number of reads mapping to each Ensemble gene was counted with htseq-count (http://www-huber.embl.de/users/anders/HTSeq/doc/count.html#count). Statistical analysis and drawing of plots was performed in R (http://www.r-project.org/) using the bioconductor package Deseq (Anders, Huber 2010) and R function gplots (http://cran.r-project.org/web/packages/gplots/index.html). Scatterplots of the RNA-seq data were drawn to illustrate differential expression between samples. A heatmap showing Euclidean distances between samples (calculated from variance stabilized transformed data) was plotted in R. For each data comparison a list of “significant differentially expressed genes” was compiled from the Deseq-result as follows: FDR<0.05, mean normalized count >10 and absolute Foldchange (FC) >1.5. Furthermore a list of genes, which are up or down regulated by *eat-2*, compared to the control (*eat-2* vs N2), but show that they are rescued in the *nhr-62;eat-2* phenotype (*nhr-62;eat-2* vs *eat-2*) was compiled and referred to as Rescued-Genes. Further analysis of “significant differentially expressed genes” and “Rescued-Genes” was performed as follows:

1. A Venn diagram was plotted in R for “significant differentially expressed genes” to show the overlap of the significant differentially expressed genes.
2. Annotation and Gene-enrichment analysis of “significant differentially expressed genes” and “Rescued-Genes” was performed with David [45], [46].
3. ReviGO analysis [63] was conducted on the DAVID GO-BP gene-enrichment result of “Rescued-Genes”. ReviGO summarizes GO-BP-IDs. The resultant network was uploaded into Cytoscape [64] for further editing and the image exported.

## Supporting Information

Figure S1 Related NHRs do not mediate DR longevity response. (A) Knockdown of *nhr-49* by RNAi does not abolish longevity of *eat-2(ad465)* mutant animals (log-rank p<0.001). (B) Percentage of animals alive on day 15 fed bacteria expressing different RNAi constructs. *nhr-49* RNAi resulted in shortevity. Knockdown of the *nhr-62* paralogs *nhr-21*, *nhr-4*, *nhr-34*, and *nhr-100* by RNAi does not suppress *eat-2;nre-1;lin-15b* longevity. (C) Median lifespan of wild-type (N2) or *eat-2(ad465)* worms fed *nhr-62*, *nhr-4*, or *nhr-21* RNAi.
(TIF)

Figure S2 Physiologic traits of *nhr-62* overexpression. (A) Pumping rates of day 1 wild-type (N2) and wild-type*(dhEx627)* worms are not different. (B) Body length of day 1 adult wild-type*(dhEx627)* are shorter than wild-type (N2) worms. (C) Oil Red O staining of wild-type (N2) and wild-type*(dhEx627)*. Oil Red O intensity in wild-type*(dhEx627)* is slightly decreased relative to wild-type (N2). ***p<0.001 by unpaired t-test. Mean (Center Line) ± SD (Box) with bars representing an outlier coefficient of 1.5.
(TIF)

Figure S3 Physiologic traits of *nhr-62* mutation. (A) Pumping rates of day 1 adult *eat-2(ad465)* and *eat-2;nhr-62* mutants are reduced relative to wild-type (N2) and *nhr-62(tm1818)* mutants (combined data from 2 independent experiments). (B) Relative body length of day 1 adult *eat-2(ad465)* and *eat-2;nhr-62* mutants are smaller than *ad libitum* controls. (C) Day 1 adult *eat-2(ad465)* and *eat-2;nhr-62* worms have reduced total progeny compared to *ad libitum* controls. *nhr-62(tm1818)* mutants have a modest but significant decrease in total hatched progeny compared to wild-type (N2) (combined data from 3 independent experiments). *p<0.05, **p<0.01, ***p<0.001 by Single Factor ANOVA with Tukey test. Mean (Center Line) ± SD (Box) with bars representing an outlier coefficient of 1.5.
(TIF)

Figure S4 Expression levels of *fat-2*, a Δ^12^-desaturase involved in PUFA synthesis. Levels of *fat-2* mRNA were significantly up-regulated under DR as measured by qPCR. Mutation of *nhr-62* partially suppressed this up-regulation, though this did not reach significance (p = n.s.). *p<0.05 by single factor ANOVA with Tukey test. Mean±SEM.
(TIF)

Figure S5 *C40H1.8* partially suppresses DR-induced longevity. (A,B) *C40H1.8* RNAi slightly reduces the lifespan of *eat-2;nre-1;lin-15b* worms relative to *gfp* RNAi fed controls (2 independent experiments). (C) Percent change in median lifespan of *eat-2;nre-1;lin-15b* relative to *nre-1;lin-15b* animals fed either *C40H1.8* RNAi or *gfp* RNAi calculated from the experiments shown in A and B.
(TIF)

Figure S6 qPCR of *C40H1.8* and homologs. Level of (A) *C40H1.7* mRNA (B) *C40H1.8* mRNA and (C) *C40H1.9* mRNA in wild-type (N2) worms fed *C40H1.8* RNAi relative to wild-type (N2) worms fed *gfp* RNAi. *p<0.05 by student's unpaired t-test. Mean±SEM.
(TIF)

Figure S7 Sequence alignment of predicted lipase *C40H1.8*. Sequence alignment of *C40H1.8* to lipase class 3 proteins in various species. Catalytic triad (CT), nucleophilic elbow (NE), dashes represent breaks in the amino acid sequence. Accession numbers are C40H1.8:NP_001021213.1, Atg15p:EEU05398.1, Os11g0299300:ABA92846.1, C40H1.9:NP_001021214.2, diacyclglycerol lipase *D. melanogaster*:ACF37118.1, diacyclglycerol lipase *M. musculus*:NP_659164.2, and diacyclglycerol lipase *H. sapiens*:NP_631918.3.
(TIF)

Figure S8 Analysis of RNA-seq data. (A) Heatmap showing Euclidean distances between RNA-seq samples (calculated from the variance stabilizing transformation of the count data). (B) Venn diagram shows the number of distinct and overlapping regulated genes from RNA-seq. (C–N) qPCR validation of RNA-seq candidates. *p<0.05, **p<0.01, ***p<0.001 by single factor ANOVA with Tukey test. Mean±SEM.
(TIF)

Table S1 Plate Aging.
(XLSX)

Table S2 BDR Aging.
(XLSX)

Table S3 Genes tested in DR lipase screen.
(XLSX)

Table S4 Genes regulated in *eat-2* versus N2.
(XLSX)

Table S5 Gene enrichment DAVID analysis of genes regulated in *eat-2* versus N2.
(XLSX)

Table S6 *nhr-62* rescued genes.
(XLSX)

Table S7 Gene enrichment DAVID analysis of rescued genes.
(XLSX)

Table S8 qPCR primers.
(XLSX)
