# Supplementary figures and images for: Dietary Restriction Induced Longevity Is Mediated by Nuclear Receptor NHR-62 in Caenorhabditis elegans
Source: PLoS Genet. 2013 Jul 25;9(7):e1003651. doi: 10.1371/journal.pgen.1003651 (PMC3723528; doi:10.1371/journal.pgen.1003651)

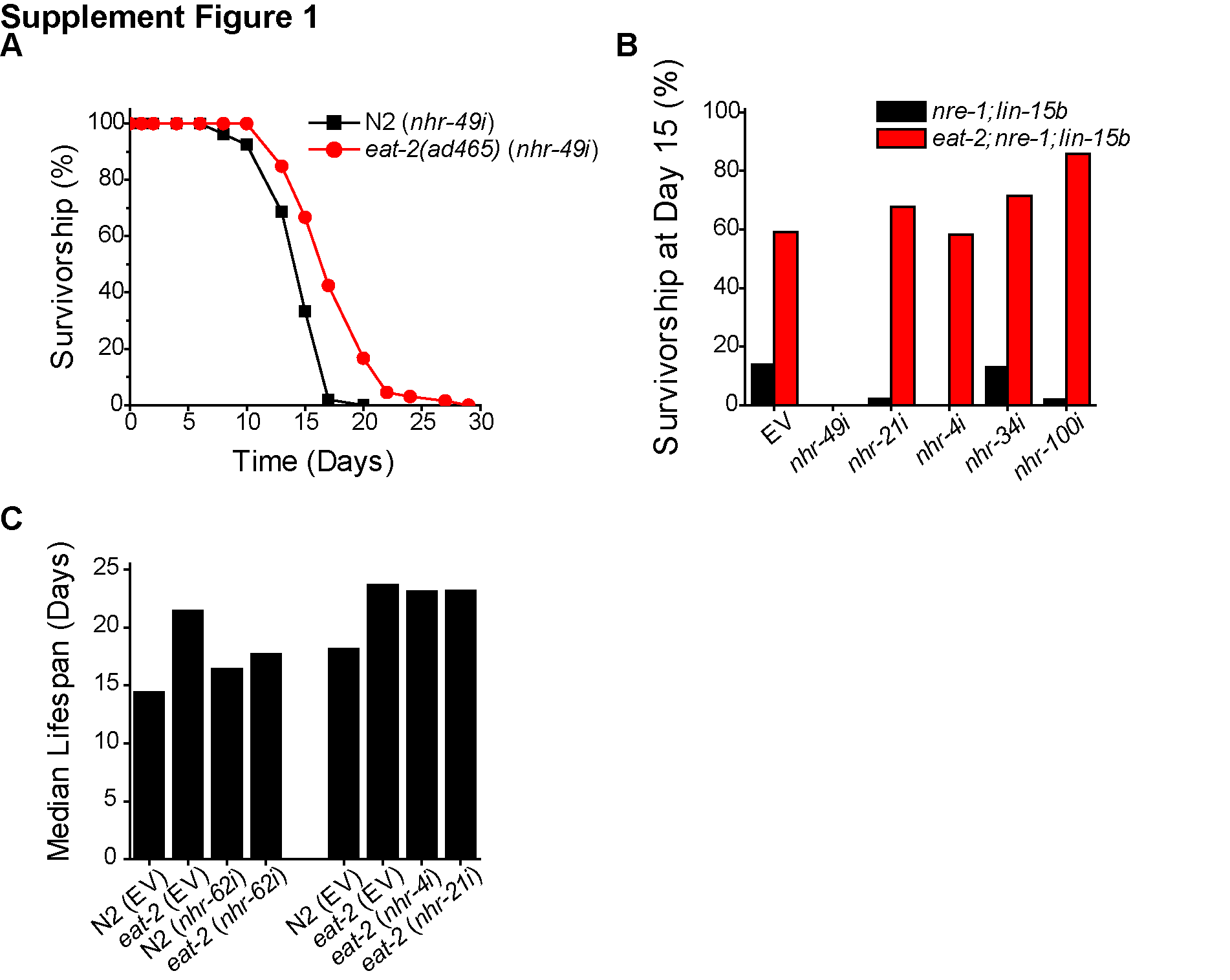

Supplement: Figure S1 — Related NHRs do not mediate DR longevity response. (A) Knockdown of nhr-49 by RNAi does not abolish longevity of eat-2(ad465) mutant animals (log-rank p<0.001). (B) Percentage of animals alive on day 15 fed bacteria expressing different RNAi constructs. nhr-49 RNAi resulted in shortevity. Knockdown of the nhr-62 paralogs nhr-21, nhr-4, nhr-34, and nhr-100 by RNAi does not suppress eat-2;nre-1;lin-15b longevity. (C) Median lifespan of wild-type (N2) or eat-2(ad465) worms fed nhr-62, nhr-4, or nhr-21 RNAi. (TIF) [file pgen.1003651.s001.tif]

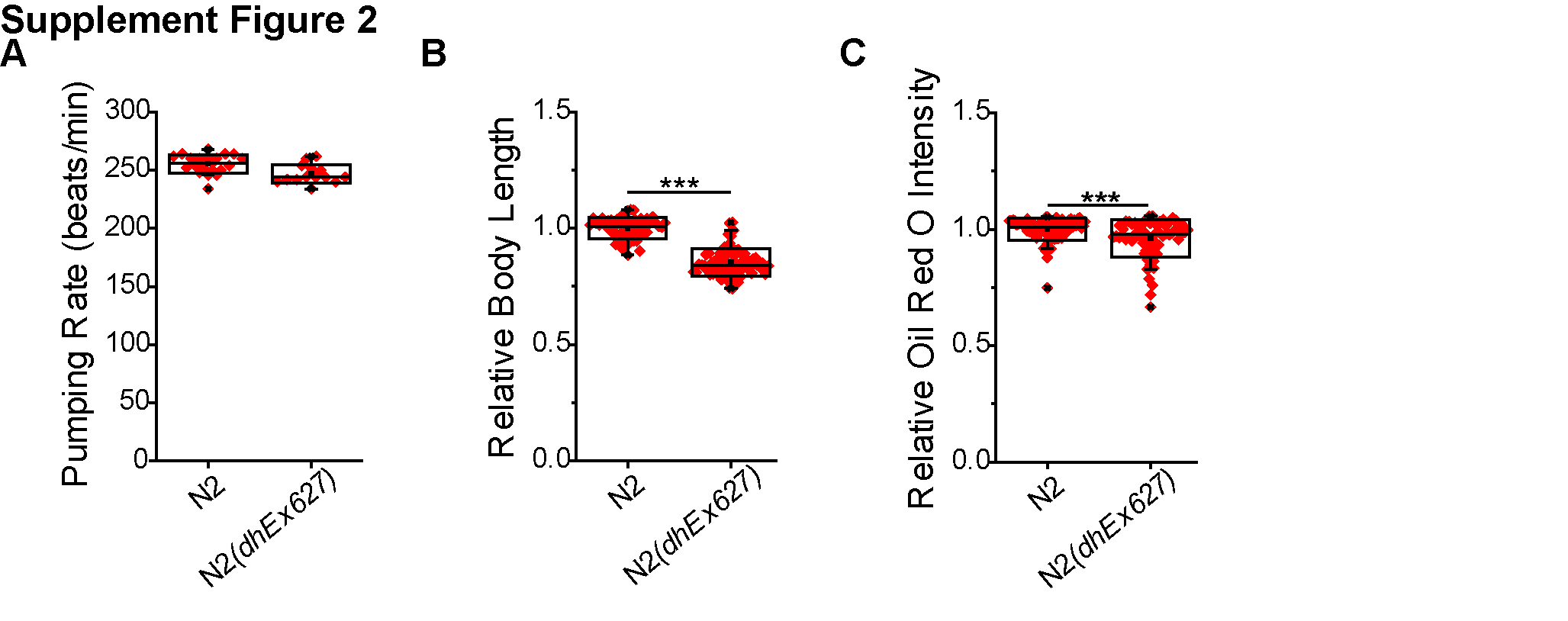

Supplement: Figure S2 — Physiologic traits of nhr-62 overexpression. (A) Pumping rates of day 1 wild-type (N2) and wild-type(dhEx627) worms are not different. (B) Body length of day 1 adult wild-type(dhEx627) are shorter than wild-type (N2) worms. (C) Oil Red O staining of wild-type (N2) and wild-type(dhEx627). Oil Red O intensity in wild-type(dhEx627) is slightly decreased relative to wild-type (N2). ***p<0.001 by unpaired t-test. Mean (Center Line) ± SD (Box) with bars representing an outlier coefficient of 1.5. (TIF) [file pgen.1003651.s002.tif]

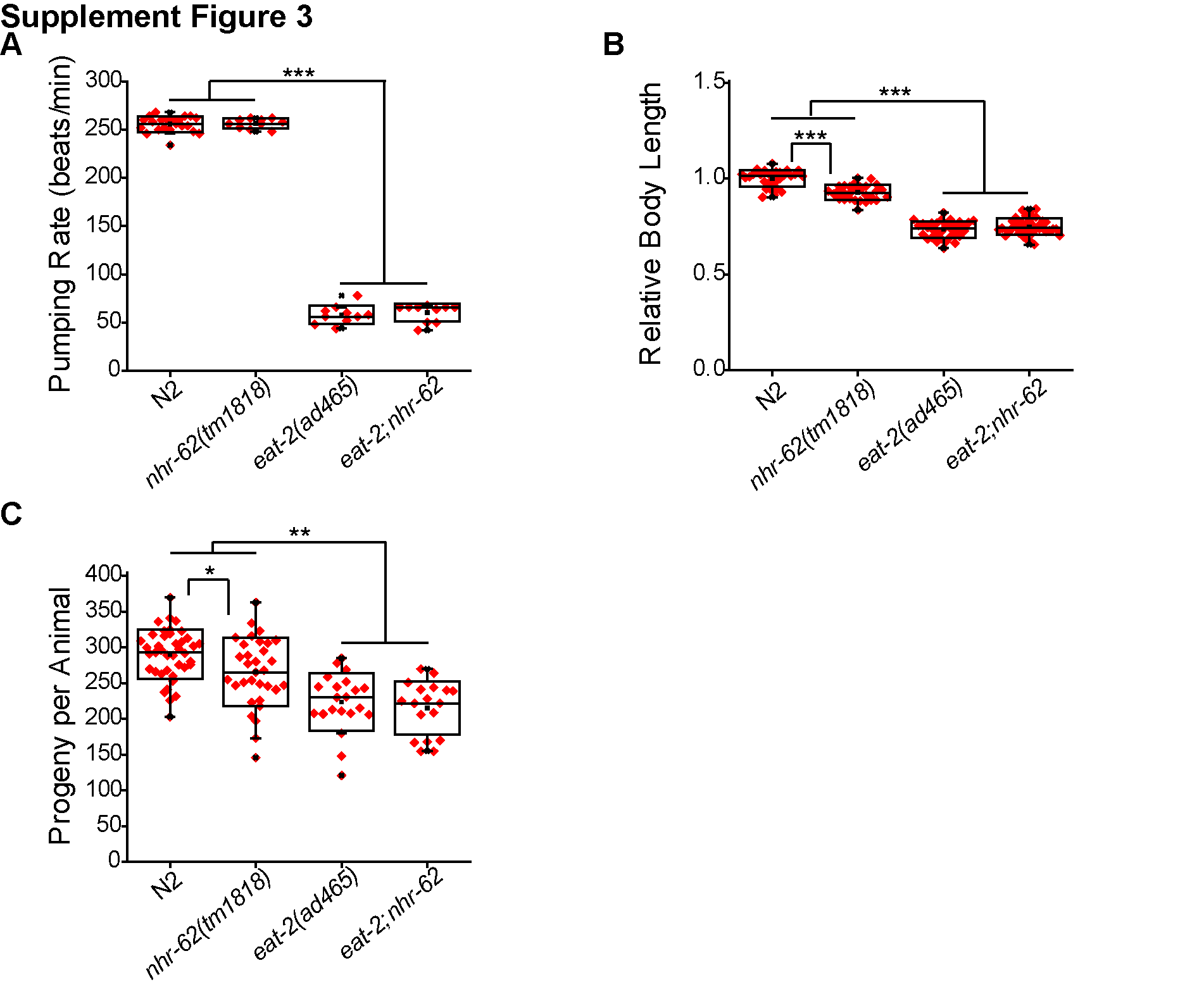

Supplement: Figure S3 — Physiologic traits of nhr-62 mutation. (A) Pumping rates of day 1 adult eat-2(ad465) and eat-2;nhr-62 mutants are reduced relative to wild-type (N2) and nhr-62(tm1818) mutants (combined data from 2 independent experiments). (B) Relative body length of day 1 adult eat-2(ad465) and eat-2;nhr-62 mutants are smaller than ad libitum controls. (C) Day 1 adult eat-2(ad465) and eat-2;nhr-62 worms have reduced total progeny compared to ad libitum controls. nhr-62(tm1818) mutants have a modest but significant decrease in total hatched progeny compared to wild-type (N2) (combined data from 3 independent experiments). *p<0.05, **p<0.01, ***p<0.001 by Single Factor ANOVA with Tukey test. Mean (Center Line) ± SD (Box) with bars representing an outlier coefficient of 1.5. (TIF) [file pgen.1003651.s003.tif]

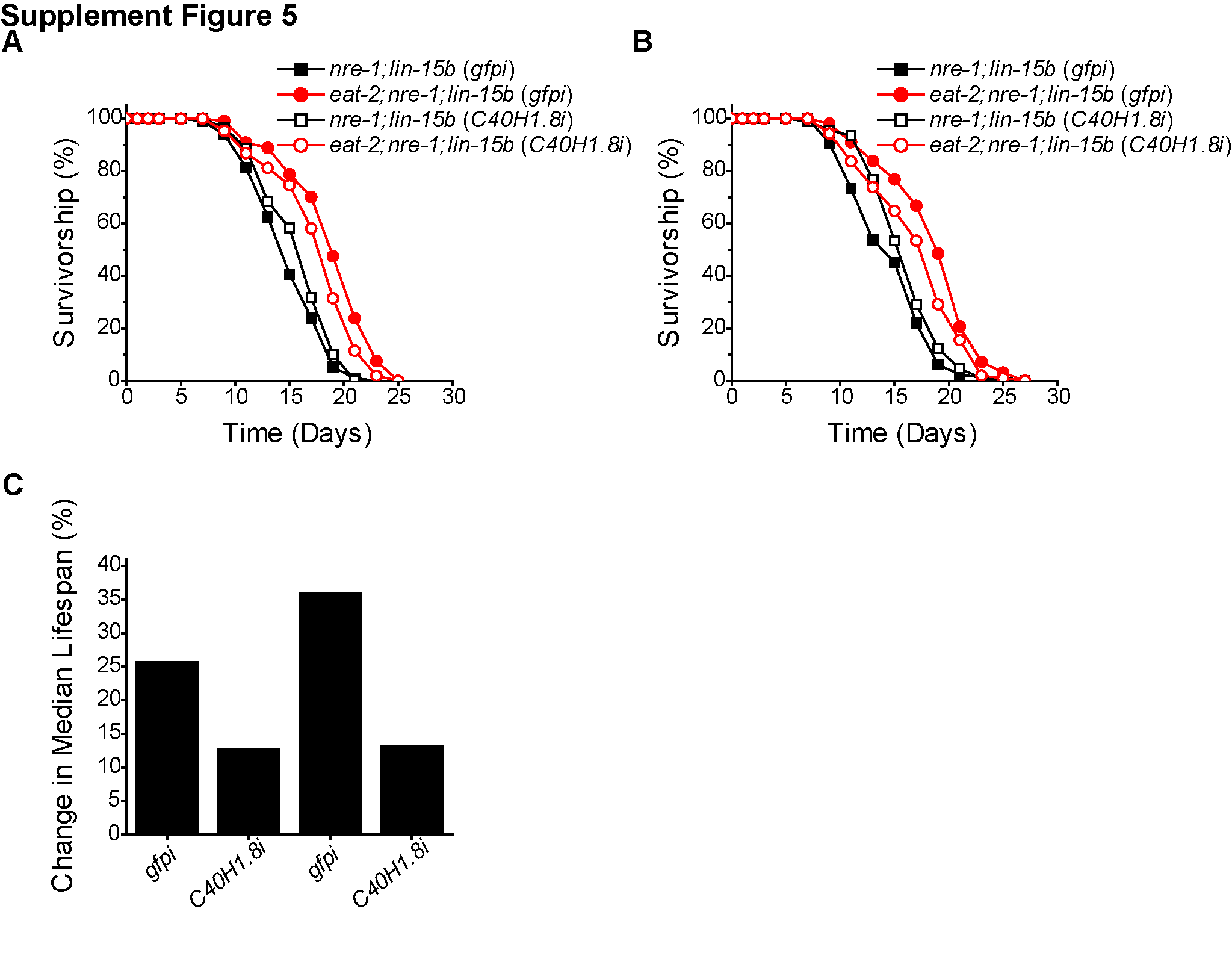

Supplement: Figure S5 — C40H1.8 partially suppresses DR-induced longevity. (A,B) C40H1.8 RNAi slightly reduces the lifespan of eat-2;nre-1;lin-15b worms relative to gfp RNAi fed controls (2 independent experiments). (C) Percent change in median lifespan of eat-2;nre-1;lin-15b relative to nre-1;lin-15b animals fed either C40H1.8 RNAi or gfp RNAi calculated from the experiments shown in A and B. (TIF) [file pgen.1003651.s005.tif]

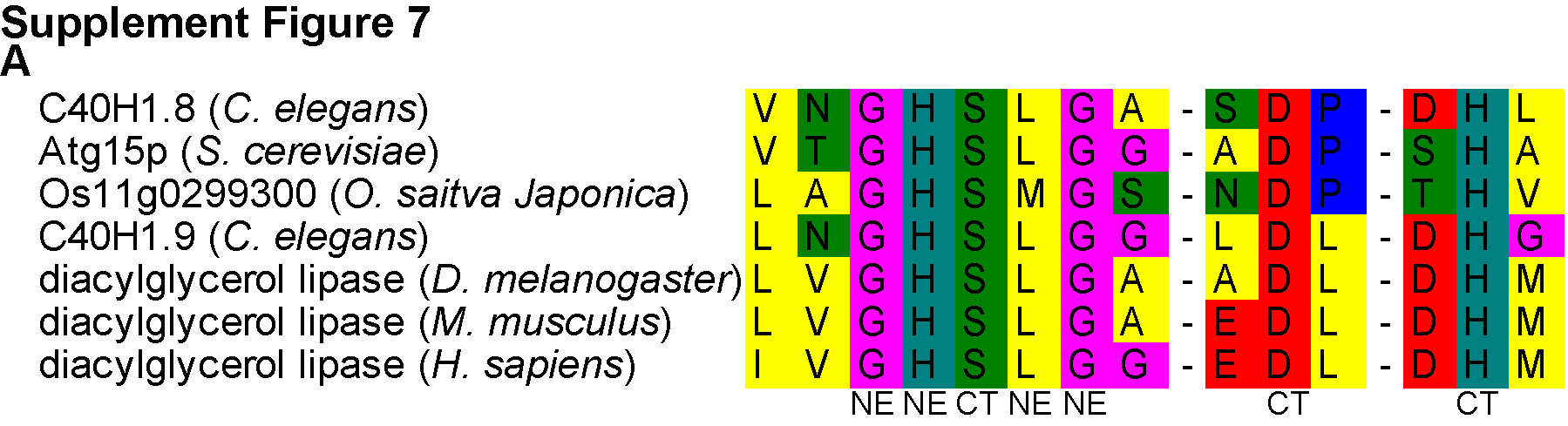

Supplement: Figure S7 — Sequence alignment of predicted lipase C40H1.8. Sequence alignment of C40H1.8 to lipase class 3 proteins in various species. Catalytic triad (CT), nucleophilic elbow (NE), dashes represent breaks in the amino acid sequence. Accession numbers are C40H1.8:NP_001021213.1, Atg15p:EEU05398.1, Os11g0299300:ABA92846.1, C40H1.9:NP_001021214.2, diacyclglycerol lipase D. melanogaster:ACF37118.1, diacyclglycerol lipase M. musculus:NP_659164.2, and diacyclglycerol lipase H. sapiens:NP_631918.3. (TIF) [file pgen.1003651.s007.tif]

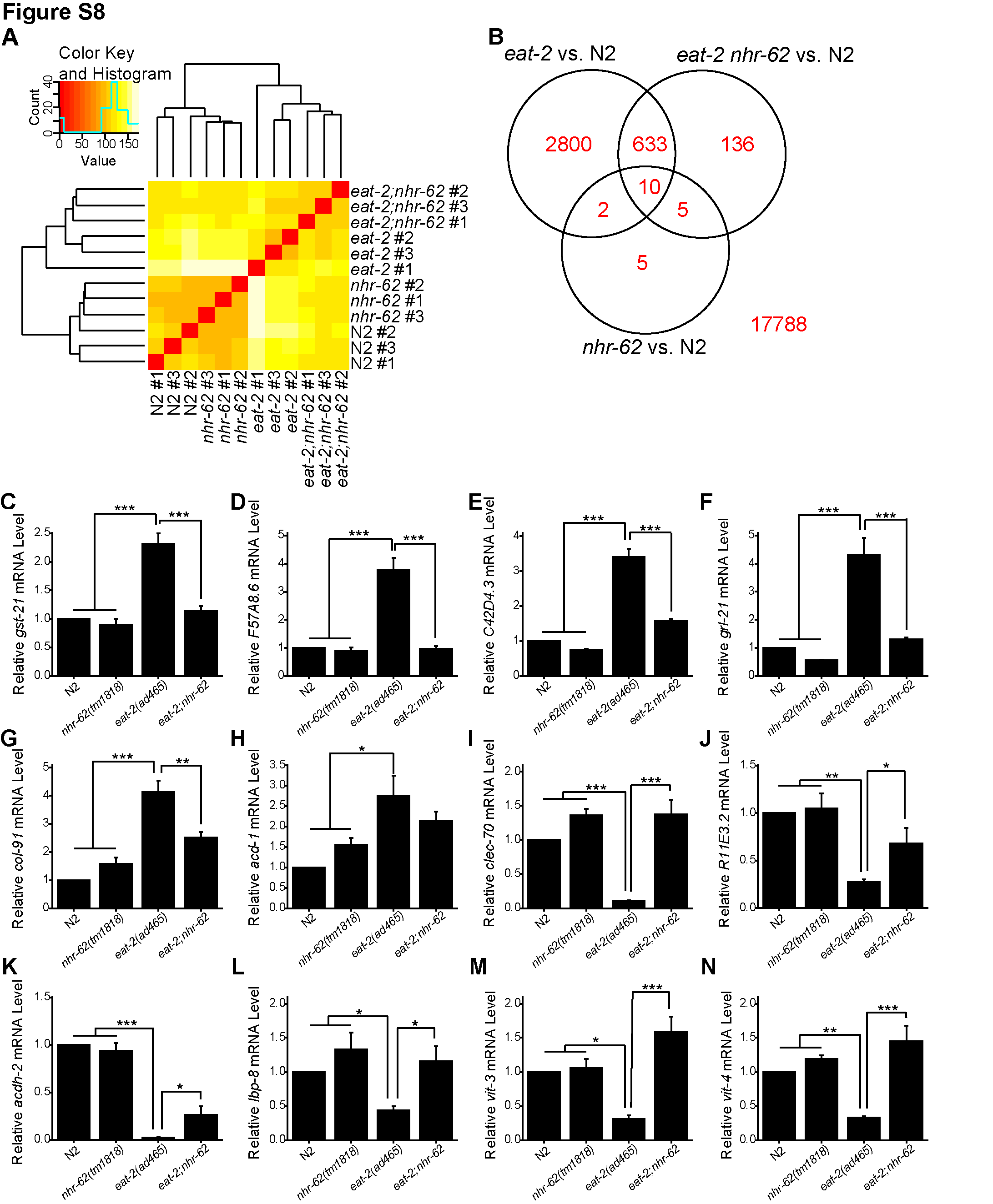

Supplement: Figure S8 — Analysis of RNA-seq data. (A) Heatmap showing Euclidean distances between RNA-seq samples (calculated from the variance stabilizing transformation of the count data). (B) Venn diagram shows the number of distinct and overlapping regulated genes from RNA-seq. (C–N) qPCR validation of RNA-seq candidates. *p<0.05, **p<0.01, ***p<0.001 by single factor ANOVA with Tukey test. Mean±SEM. (TIF) [file pgen.1003651.s008.tif]
